# Maternal Smoking During Pregnancy Predicts Adult Offspring Cardiovascular Risk Factors – Evidence from a Community-Based Large Birth Cohort Study

**DOI:** 10.1371/journal.pone.0041106

**Published:** 2012-07-19

**Authors:** Abdullah A. Mamun, Michael J. O'Callaghan, Gail M. Williams, Jake M. Najman

**Affiliations:** 1 School of Population Health, University of Queensland, Brisbane, Australia; 2 Child Development and Rehabilitation Services, Mater Children's Hospital, Brisbane, Australia and School of Medicine, University of Queensland, Brisbane, Australia; 3 School of Social Science, University of Queensland, Brisbane, Australia; Tulane School of Public Health and Tropical Medicine, United States of America

## Abstract

**Background:**

Maternal smoking during pregnancy is associated with offspring obesity. However, little is known about whether maternal smoking in pregnancy predicts other offspring cardiovascular risk factors including waist circumference (WC), waist-hip-ratio (WHR), pulse rate (PR), systolic (SBP), and diastolic blood pressure (DBP).

**Methods:**

We studied a sub-sample of 2038 (50% males) young adults who were born in Brisbane, Australia to investigate the prospective association of maternal smoking during pregnancy with young adult cardiovascular risk factors. We compared offspring mean BMI, WC, WHR, SBP, DBP and PR and the risk of being overweight and obese at 21 years by three mutually exclusive categories of maternal smoking status defined as never smoked, smoked before and/or after pregnancy but not in pregnancy or smoked during pregnancy and other times.

**Results:**

Offspring of mothers who smoked during pregnancy had greater mean BMI, WC, WHR and PR and they were at greater risk of being obese at 21 years compared to offspring of those mothers who never smoked. The mean of these risk factors among those adult offspring whose mothers stopped smoking during pregnancy, but who then smoked at other times in the child's life, were similar to those mothers who never smoked. These results were independent of a range of potential confounding factors.

**Conclusion:**

The findings of this study suggest a prospective association of maternal smoking during pregnancy and offspring obesity as well as PR in adulthood, and reinforce the need to persuade pregnant women not to smoke.

## Introduction

Meta-analysis have shown that maternal smoking during pregnancy is consistently associated with overweight and obesity in offspring from ages 3–33 years [1], [2]. Further, infants of mothers who smoked during pregnancy have a greater catch-up growth [3] and such growth acceleration has been associated with increased cardiovascular risk [4], [5]. Overweight and obesity status in offspring predict future cardiovascular risk [6]. Most of the studies which have examined the prospective associations between maternal smoking during pregnancy and offspring overweight and obesity are based on the measured/self-reported body mass index (BMI) and its categories. Relatively less is known about longer term relationships and whether maternal smoking in pregnancy predicts offspring cardiovascular risk factors including central obesity, pulse rate (PR) and blood pressure (BP) in adult offspring.

A few studies have investigated the association between prenatal smoking exposure and high BP in the offspring [7]–[11], with some [8]–[10] but not all [7], [11] studies showing a positive association. Most studies of offspring of smokers have been conducted in pre-pubertal children [9], [10], [12]–[17] but only a few in adults [18], [19]. Most of these studies have used only BMI to indicate overweight or obesity status in offspring, with few examining other measures of adiposity such as lean mass [14] or waist circumference (WC) [11]. Further, none of the studies have examined the association of maternal smoking during pregnancy with future offspring PR, which is also receiving recent attention as a cardiovascular risk factor [20]. Recently, Power et al found that adults who had been exposed to tobacco in utero had a higher adult BMI, WC, BP and cholesterol. However, except for BMI and WC, all the other associations were not statistically significant after adjustment for postnatal confounders [11]. Hence, further research on the long-term associations between prenatal smoking exposure and cardiovascular disease risk is required to establish whether any observed associations may be due to intrauterine or postnatal effects.

In a previous study we found that maternal smoking during pregnancy was associated with future adolescent BMI and obesity [21]. Apart from this study none of the previous studies have compared the offspring of mothers who smoked during pregnancy with the offspring of mothers who were never smokers and mothers who smoked at other times before or after pregnancy but not during pregnancy. The aims of this study are to examine (i) whether the magnitude of the association between maternal smoking during the pregnancy and adolescent obesity diminished or strengthened or remains the same with age (i.e. from 14 to 21 years) and (ii) whether maternal smoking during pregnancy is associated with young adults' BMI, WC, BP and PR at 21 years of age using an Australian community based large birth cohort study.

## Materials and Methods

### Participants

Data came from the Mater-University of Queensland Study of Pregnancy (MUSP), which is a prospective study of 7,223 mother-offspring pairs. Mothers received antenatal care at a major public hospital in Brisbane between 1981 and 1983 and delivered a live singleton child who was not adopted out before leaving hospital [22], [23]. Mothers and children have been followed-up prospectively with maternal questionnaires being administered at first antenatal clinic visit (FCV), 3–5 days post-delivery, 6 months, 5, 14 and 21 years after birth. At 21 years, mothers and young adults completed a self administered questionnaire and MUSP research staff undertook a physical assessment of a subsample of the participants including the measurements of height, weight and BP. In this paper, analyses were restricted to the 2038 mothers for whom prospective data were available on self-reported smoking status from pre-pregnancy to 14 years post delivery and where data were available on measured height, weight, WC, WHR, BP and PR for offspring at 21 years. Informed consent from the mothers was obtained at all data collection phases of the study, and from the youth at 14 and 21 years. Ethics committees at the Mater Hospital and the University of Queensland approved each phase of the study. Full details of the study participants and measurements have been previously reported [22], [23].

In general, participants lost to follow-up were more likely to be males and of Asian and Aboriginal/Torres Strait Islander background (all p-values<0.001). Their mothers were more likely to be teenagers, less educated, single or cohabitating, have three or more children, used tobacco and alcohol during pregnancy and be anxious and depressed at their first antenatal visit [22].

### Maternal smoking during pregnancy

Smoking status was assessed at the FCV when the average duration of gestation was 18-weeks, and then again 3–5 days after delivery and at 6 months, 5, 14 and 21 years of follow-up. The measurement of maternal smoking from pre-pregnancy to 14 years post-delivery has been previously reported [21]. In the main analysis, we grouped maternal smoking status into three mutually exclusive categories: 1) never smoked (responded “no” at all stages of the study); 2) smoked throughout pregnancy (responded “yes” to the question on smoking at the first clinical visit and/or “yes” to the question on smoking in the third trimester); and 3) smoked before and/or after pregnancy but not during pregnancy (responded “no” to the questions on smoking at the first clinical visit and during the third trimester but “yes” to questions on smoking before pregnancy and/or at any stage of follow-up).

### Measurement of outcomes

At the 21 year follow-up, offspring's height was measured without shoes using a portable stadiometer to the nearest centimetre. Weight was measured in light clothing with a scale accurate to 0.2 kg. Two measures of weight and height were taken and the mean of these two measures was used in all analyses. BMI (weight in kilograms divided by the square of height in meters i.e. kg/m^2^) was categorized into normal (<25 kg/m^2^), overweight (25–29 kg/m^2^) and obese (> = 30 kg/m^2^) using the WHO classification of BMI cut-offs [24]. WC (in cm) and WHR were measured at the same time using a standard protocol. WC was categorized for males as follows: <94 cm as normal, 94–<102 cm as overweight and 102 or more cm as obese; and for females as follows: <80 cm as normal, 80–<88 cm as overweight and 88 or more cm as obese [25]. WHR was categorized for males as normal if WHR<0.90, WHR: 0.90–<1.00 as overweight and WHR> = 1.00 as obese. For females as normal if WHR<0.80, overweight if WHR: 0.80–<0.85 and WHR> = 85 as obese [25]. Both systolic blood pressure (SBP in mmHg) and DBP (mmHg) were assessed at age 21 years with the participant rested and their arm supported at chest level. Two readings were taken five minutes apart, using the OMRAN HEM-703C automatic BP device and with the appropriate cuff size for arm circumference. Young adults were classified as normotensive (SBP<120 and DBP<80), pre-hypertensive (measured SBP 120–140 and DBP 80–90) and hypertensive (SBP>140 or DBP>90). Using the same machine two readings of PR (beats per minute) were taken five minutes apart. The mean of the two measurements for SBP, DBP and PR were used in all analyses.

### Measurements of confounders

The following maternal characteristics during pregnancy, reported at FCV, were considered to be potential confounding factors on the basis of their association with maternal smoking during pregnancy and offspring cardiovascular risk factors: maternal age, maternal education (did not complete secondary school, completed secondary school, completed further/higher education) and maternal pre-pregnancy BMI. Maternal pre-pregnancy BMI was calculated based on the maternal measured height at pregnancy and self-reported pre-pregnancy weight, which was recorded at the study initiation from maternal questionnaires. The child's characteristics that were considered as potential confounders or mediators were birth weight (measured in grams), breastfeeding (categorized into three groups: never, <4 months, > = 4 months, recorded at 6-month follow-up), child gender (male or female), childhood diet and physical activity patterns. Childhood diet and child physical activity data were obtained from maternal report at the 14-years follow-up. Mother's were asked to report the frequency of their child's consumption of fast food, salad, soft drinks and red meat (all with response options of rarely or never, at least 2 or 3 times a week, most days) and to report the amount of time her child spent watching television (<1 hour per day, 1 to <3 hour per day, 3 to <5 hour per day and 5 or more hour per 1day) and the amount of time they spent on sports or exercise (4–7 days per week; 0–3 days per week).

### Statistical analyses

We examined the association between maternal smoking categorised into different mutually exclusive groups and offspring cardiovascular risk factors at 21 years post-partum. The mean BMI, WC, WHR, SBP, DBP and PR by three mutually exclusive groups of maternal smoking were compared by one-way analysis of variance and computing an F-test when the outcomes were continuous assessments of cardiovascular risk factors (Table 1). Statistical evidence for a difference in effect between males and females was assessed by computing a likelihood ratio test of the interaction with sex. As we found no statistical evidence that the effect differed between the sexes, results are presented for males and females combined.

**Table 1:** Young adult's mean (SD) BMI, WC, WHR, SBP, DBP and PR at 21 years by three mutually exclusive categories of maternal smoking status during pregnancy (N = 2038).

	Maternal smoking status			
Cardiovascular risk factors at 21 yrs	Never smoked	Smoked before and/or	Smoked during	p-value*
		after pregnancy	pregnancy	
**Body mass index**	23.82(4.49)	23.62(4.12)	24.32(4.68)	0.029
**Waist circumference**	80.83(11.62)	80.66(10.64)	82.22(12.06)	0.029
**Waist-hip-ratio**	0.82(0.77)	0.82(0.70)	0.83(0.08)	0.008
**Systolic blood pressure**	116.06(14.74)	115.69(14.37)	116.51(14.05)	0.696
**Diastolic blood pressure**	67.86(8.56)	67.70(8.27)	67.12(8.30)	0.173
**Pulse rate**	74.96(11.82)	74.68(11.32)	76.40(11.73)	0.021

* **We used an F test for comparing mean differences by three mutually exclusive categories of the maternal smoking status.**

As in the crude analyses, we found both SBP and DBP and its categorization (i.e. normotensive, pre-hypertensive or hypertensive) were not associated with the maternal smoking status; we did not include them in the further multivariable analyses. A series of multiple linear regression models (see footnotes to tables 1 and 2) were used to determine the mean difference in BMI, WC, WHR and PR by maternal smoking status in the three mutually exclusive categories taking into account potential confounding or mediating factors. Similarly, a series of multinomial regression models were used to assess the association between maternal smoking status and their offspring being overweight and obese at age 21 (table 3) by the BMI, WC and WHR categories.

**Table 2:** Mean difference (95% Confidence Interval) in BMI, WC, WHR and PR of young adults by three mutually exclusive categories of maternal smoking status, adjusting for potential confounding factors (N = 1820).

	Maternal Smoking Status				
Model No	Never Smoked	Smoked before or after pregnancy but not during pregnancy		Smoking during pregnancy	
	Mean difference in BMI (95% Confidence interval)				
Model 1	0	−0.17	(−0.84,0.50)	0.54	(0.09,0.98)
Model 2	0	−0.23	(−0.90,0.44)	0.52	(0.08,0.96)
Model 3	0	−0.10	(−0.75, 0.54)	0.71	(0.28, 1.14)
Model 4	0	−0.08	(−0.73, 0.56)	0.73	(0.28, 1.17)
	Mean difference in WC (95% Confidence interval)				
Model 1	0	−0.32	(−2.06, 1.41)	1.37	(0.22, 2.51)
Model 2	0	−0.12	(−1.75, 1.51)	1.47	(0.40, 2.54)
Model 3	0	0.24	(−1.36, 1.84)	1.98	(0.91, 3.04)
Model 4	0	0.24	(−1.34, 1.84)	2.12	(1.02, 3.22)
	Mean difference in WHR (95% Confidence interval)				
Model 1	0	0	(−0.01,0.02)	0.01	(0.00,0.02)
Model 2	0	0.01	(−0,00.02)	0.01	(0.01,0.02)
Model 3	0	0.01	(−0.00.02)	0.01	(0.01,0.02)
Model 4	0	0.01	(−0.00 0.02)	0.01	(0.01,0.02)
	Mean difference in PR (95% Confidence interval)				
Model 1	0	−0.10	(−1.85, 1.65)	1.29	(0.14,2.44)
Model 2	0	−0.15	(−1.90, 1.60)	1.27	(0.12, 2.42)
Model 3	0	−0.24	(−2.06, 1.53)	1.15	(−0.01, 2.33)
Model 4	0	−0.27	(−2.04, 1.49)	0.98	(−0.24, 2.20)

Model 1: unadjusted.

Model 2: adjusted for age and sex.

Model 3: model 2 plus maternal age at first clinic visit, maternal education and maternal pre-pregnancy body mass index.

Model 4: model 3 plus birth weight (in grams), breastfeeding, maternal report of the child's consumption of fast food, salad, soft drinks, and red meat, and maternal report of the child's amount of television watching and participation in sports and exercise.

**Table 3:** Adjusted odds ratio (95% confidence interval) for overweight and obesity at age 21 years among offspring of mothers who smoked throughout pregnancy or smoked before or after pregnancy but not during pregnancy as compared with the offspring of mothers who never smoked (reference category).

	Maternal Smoking Status				
Model No	Never Smoked	Smoked before or after pregnancy but not during pregnancy		Smoking during pregnancy	
	BMI: OR (95% CI)				
**Model 1**					
Normal	1	1	1	1	1
Overweight	1	0.86	(0.58, 1.26)	1.18	(0.93, 1.51)
Obese	1	0.79	(0.46,1.37)	1.38	(1.01, 1.90)
**Model 2**					
Normal	1	1	1	1	1
Overweight	1	0.84	(0.57, 1.24)	1.18	(0.93, 1.51)
Obese	1	0.76	(0.44, 1.32)	1.37	(1.00, 1.89)
**Model 3**					
Normal	1	1	1	1	1
Overweight	1	0.86	(0.58, 1.28)	1.25	(0.96, 1.61)
Obese	1	0.85	(0.48, 1.49)	1.60	(1.14, 2.25)
**Model 4**					
Normal	1	1	1	1	1
Overweight	1	0.87	(0.58, 1.29)	1.28	(0.98, 1.67)
Obese	1	0.85	(0.48,1.51)	1.56	(1.10, 2.22)
	WC: OR (95% CI)				
**Model 1**					
Normal	1	1	1	1	1
Overweight	1	1.00	(0.65,1.55)	1.20	(0.91, 1.60)
Obese	1	0.81	(0.48,1.35)	1.29	(0.96, 1.75)
**Model 2**					
Normal	1	1	1	1	1
Overweight	1	0.95	(0.61, 1.48)	1.19	(0.89,1.58)
Obese	1	0.75	(0.45, 1.27)	1.28	(0.94,1.73)
**Model 3**					
Normal	1	1	1	1	1
Overweight	1	1.02	(0.65,1.60)	1.29	(0.96, 1.75)
Obese	1	0.79	(0.46, 1.35)	1.44	(1.05,1.99)
**Model 4**					
Normal	1	1	1	1	1
Overweight	1	1.02	(0.65,1.61)	1.31	(0.96, 1.79)
Obese	1	0.79	(0.46,1.36)	1.43	(1.02,2.00)
	WHR: OR (95% CI)				
**Model 1**					
Normal	1	1	1	1	1
Overweight	1	1.32	(0.92,1.89)	1.27	(1.00, 1.62)
Obese	1	0.93	(0.51, 1.69)	1.72	(1.23, 2.41)
**Model 2**					
Normal	1	1	1	1	1
Overweight	1	1.32	(0.92, 1.89)	1.27	(1.00, 1.62)
Obese	1	0.85	(0.46, 1.57)	1.71	(1.21, 2.43)
**Model 3**					
Normal	1	1	1	1	1
Overweight	1	1.34	(0.93, 1.92)	1.27	(1.00, 1.64)
Obese	1	0.89	(0.48, 1.65)	1.87	(1.30, 2.70)
**Model 4**					
Normal	1	1	1	1	1
Overweight	1	1.34	(0.93, 1.93)	1.21	(0.93, 1.57)
Obese	1	0.89	(0.48, 1.66)	1.79	(1.23, 2.60)

Model 1: unadjusted

Model 2: adjusted for age and sex

Model 3: model 2 plus maternal age at first clinic visit, maternal education and maternal pre-pregnancy body mass index

Model 4: model 3 plus birth weight (in grams), breastfeeding, maternal report of the child's consumption of fast food, salad, soft drinks, and red meat, and maternal report of the child's amount of television watching and participation in sports and exercise.

To test whether the association between maternal smoking status and offspring PR was mediated by offspring BMI at 21 years, the multivariable model was additionally adjusted for 21 years BMI. We also performed the Sobel-Goodman mediation tests [26].

All analyses were undertaken using Stata version 11.0 (Stata inc., Texas).

## Results

Of 2038 participants for whom we have measured cardiovascular risk factors and maternal self-reported smoking status over 21 years post-partum, 37.05% of mothers smoked during pregnancy and at other time, 11.19% smoked before/after pregnancy but not during pregnancy and the rest (51.77%) never smoked. The mean (SD) BMI, WC, WHR, SBP, DBP and PR for those young adults were 23.98 (4.52) kg/m^2^, 81.33(11.70) cm, 0.83 (0.08), 116.18(14.44) mmHg, 67.57(8.43) mmHg and 75.46(11.75) BPM, respectively.

Table 1 shows the mean (SD) values of BMI, WC, WHR, SBP, DBP and PR at age 21 years by maternal smoking categories. Except for SBP and DBP, we found that the offspring of mothers who smoked during pregnancy had significantly greater mean BMI, WC, WHR and PR while offspring of mothers who smoked at other times but not in pregnancy had similar mean BMI, WC, WHR and PR to offspring of never smoking mothers.

Table 2 shows the mean difference in BMI, WC, WHR and PR at age 21 years between offspring in each smoking group and offspring in the reference group (never smoking), with adjustment for potential confounders or mediators in a series of multiple regression models. Results are presented for the 1820 young adults with complete data on all variables included in any of the multivariable models. In the age- and sex-adjusted model (model 2), we found that the offspring of mothers who smoked during pregnancy had a BMI, WC, WHR and PR that was higher by 0.52 kg/m^2^ (95% confidence interval: 0.08, 0.96), 1.47 (0.40, 2.54) cm, 0.01 (0.01, 0.02), and 1.27 (0.12, 2.44) respectively, on average, than that of offspring of mothers who never smoked. The BMI, WC, WHR and PR of offspring whose mothers smoked at any other time but not during pregnancy was the same as that for offspring of mothers who never smoked. Except, for PR, adjustment for covariates did not substantively alter these associations. The association between maternal smoking status and offspring PR at 21 years was partially mediated by the 21 year BMI.

Unadjusted and adjusted odds of becoming overweight and obese at 21 years by the maternal smoking status are presented in table 3. In the age- and sex-adjusted model, the offspring of women who smoked throughout pregnancy had increased odds of being obese (using BMI cut-off: odds ratio 1.37; 95% confidence interval: 1.00, 1.89; WC cut-off: OR 1.28: 0.94, 1.70 and WHR cut-off: OR 1.71; 1.21, 2.43) at age 21 years (table 3, model 2). Adjustment for a range of potentially confounding or mediating factors did not substantially alter these associations, except the adjustment for maternal pre-pregnancy BMI which strengthened the overall association.

When we repeated the analyses after removing the 62 women who reported smoking before the birth of their child but at no other time, the results did not differ from those presented here. Similarly, when we repeated the analyses after removing the 27 women who reported smoking during their child's pregnancy but then quitting afterward, the results were not altered.

## Discussion

In this study, based on the prospective follow-up of a mother and-child cohort, we found that young adult BMI, WC, WHR, PR and the prevalence of obesity were greater among offspring whose mothers had smoked during pregnancy than among offspring whose mothers had never smoked. We also found mean BMI, WC, WHR, PR and the prevalence of obesity among young adults whose mothers smoked before and/or after pregnancy but not during pregnancy were similar to these outcomes in young adults whose mothers had never smoked. Except for the PR, these associations were robust after adjustment for a variety of potentially confounding or mediating factors, including maternal pre-pregnancy BMI, childhood dietary patterns, television watching, and participation in sports. Both SBP and DBP were not directly associated with maternal smoking during pregnancy. Findings of this study may provide some evidence for a direct association of maternal smoking in utero predicting later development of cardiovascular risk factors including increased adiposity, obesity and PR in offspring.

This prospective follow-up study of mother-offspring pairs, extends the existing evidence on the relation between maternal smoking in pregnancy and later obesity in childhood [1], [2] to a persistence of the effect into adult cardiovascular risk factors including obesity, adiposity and PR in young adulthood. The strength and magnitude of the association of maternal smoking in pregnancy with offspring BMI (mean difference is 0.52 in age and sex adjusted model) and obesity (OR 1.37 in age and sex adjusted model) at 21 years are similar to a previous study where we examined this relationship for adolescents (mean difference in BMI was 0.56 and OR for obesity 1.42 in age and sex adjusted model) [21]. We have added to the findings of other studies by adjusting for a greater range of potentially explanatory factors, including offspring diet, physical activity levels and TV watching, and by being able to compare the offspring of mothers who smoked during pregnancy with the offspring of both never smokers and mothers who smoked at other times before or after pregnancy but not during pregnancy.

Though findings from this study support a prospective association between future obesity, adiposity, PR and maternal smoking in pregnancy, this is an observational study and other explanations for the findings need to be considered. For example, those mothers who cease smoking in pregnancy may be more health conscious in relation to care of their child and may have a greater commitment to cultivating a healthy lifestyle. That is maternal attitude to health and unmeasured lifestyle differences provide a possible alternative explanation of our findings. In our study, adjustment for maternal reporting of children's diet, physical activity and TV watching did not substantively alter the association, but we cannot rule out residual confounding including maternal socioeconomic position or social class and offspring genetic predisposition.

Overall, the association between maternal smoking during pregnancy with offspring BMI, WC and WHR were stronger than with offspring BP and PR. Although BP was not directly associated with maternal pregnancy smoking, the direction and magnitude of effect were similar to BMI. Maternal smoking may lead to offspring BMI or obesity which indirectly influences offspring BP and PR. We conducted Sobel-Goodman tests [26] to examine whether offspring BMI at 21 years mediated the association between maternal pregnancy smoking and offspring 21year PR. This test showed a small indirect effect of 0.10 (direct effect 1.20 and total effect 1.30). The proportion of total effect mediated was 0.07, though it was statistically significant (p-value<0.05).

Cigarettes contain multiple compounds [27], [28] other than nicotine, so establishing a precise mechanism linking maternal smoking in pregnancy and later cardiovascular risk in offspring is difficult. Neonatal exposure to nicotine however has been linked in animal studies to increased post natal weight gain and adiposity [29]. Other studies [30], [31] suggest that nicotine may also affect neurotransmitter levels and in-utero hypothalamic development and function including longer term effects on appetite control. Further studies using animal models may facilitate an understanding of these mechanisms, the possible role of other chemicals contained in cigarettes, and causal mechanisms linking pregnancy cigarette exposure to later cardiovascular risk.

Mothers not attending the 21 year follow-up were more likely to be non white and to have a lower level of education and family income than participants. Attrition is unlikely to result in significant attenuation of study findings. Children of mothers who smoke during pregnancy are more likely to have adverse physical and mental health outcomes [22], [23] and it is unlikely that the cigarette exposure in pregnancy and cardiovascular risk would be non existent or in the opposite direction in non attendees to that found in study children. The prevalence of overweight and obesity of 34% in the MUSP study offspring at 21 years was identical to the National Nutrition Survey. Self reporting of cigarette smoking in pregnancy may be subject to bias. Given the potential social stigma associated with smoking, this is most likely to lead to an underreporting and for smoking mothers to be included with non smoking mothers. This would lead to an attenuation of our results with the real effect size being larger than that reported in this study.

Few women ceased smoking during pregnancy with the largest study groups being those who never smoked and those smoking at all study times. This suggests a need to focus interventions not only on cigarette use in pregnancy but also primary prevention at the time of smoking initiation.

Using longitudinal analyses of a mother and-offspring cohort, we found that the young adult offspring of mothers who reported having smoked during pregnancy were more likely to have higher BMI, WC, WHR and higher PR than those whose mothers did not smoke during pregnancy. Our findings suggest the possibility of a direct association of maternal smoking in pregnancy on young adult cardiovascular risk factors. These provide yet another incentive for pregnant women to be persuaded not to smoke and for young women to be encouraged to never take up smoking.

## References

[1] Ino T (2010). Maternal smoking during pregnancy and offspring obesity: Meta-analysis.. Pediatr In.

[2] Oken E, Levitan EB, Gillman MW (2008). Maternal smoking during pregnancy and child overweight: systematic review and meta-analysis.. Int J Obes.

[3] Nafstad P, Jaakkola JJK, Hagen JA, Pedersen BS, Qvigstad E (1997). Weight gain during the first year of life in relation to maternal smoking and breast feeding in Norway.. J Epidemiol Community Health.

[4] Oken E, Gillman MW (2003). Fetal origins of obesity.. Obes Res.

[5] Ong KKL (2000). Association between postnatal catch-up growth and obesity in childhood: prospective cohort study.. BMJ.

[6] Vanhala M (1999). Childhood weight and metabolic syndrome in adults.. Ann Med.

[7] Bergel E, Haelterman E, Belizan J, Villar J, Carroli G (2000). Perinatal factors associated with blood pressure during childhood.. Am J Epidemiol.

[8] Blake KV, Gurrin LC, Evans SF, Beilin LJ, Landau LI (2005). Maternal cigarette smoking during pregnancy, low birth weight and subsequent blood pressure in early childhood.. Early Hum Dev.

[9] Morley R, Payne CL, Lister G, Lucas A (1995). Maternal smoking and blood pressure in 7.5 to 8 year old offspring.. Arch Dis Child.

[10] Oken E, Huh SY, Taveras EM, Rich-Edwards JW, Gillman MW (2005). Associations of maternal prenatal smoking with child adiposity and blood pressure.. Obes Res.

[11] Power C, Atherton K, Thomas C (2010). Maternal smoking in pregnancy, adult adiposity and other risk factors for cardiovascular disease.. Atherosclerosis.

[12] Chen AM, Pennell ML, Klebanoff MA, Rogan WJ, Longnecker MP (2006). Maternal smoking during pregnancy in relation to child overweight: follow-up to age 8 years.. Int J Epidemiol.

[13] Fasting MH, Oien T, Storro O, Nilsen TIL, Johnsen R (2009). Maternal smoking cessation in early pregnancy and offspring weight status at four years of age. A prospective birth cohort study.. Early Hum Dev.

[14] Leary SD, Smith GD, Rogers IS, Reilly JJ, Wells JCK (2006). Smoking during pregnancy and offspring fat and lean mass in childhood.. Obesity.

[15] Suzuki K, Ando D, Sato M, Tanaka T, Kondo N (2009). The association between maternal smoking during pregnancy and childhood obesity persists to the age of 9–10 Years.. J Epidemiol.

[16] Toschke AM, Koletzko B, Slikker W, Hermann M, von Kries R (2002). Childhood obesity is associated with maternal smoking in pregnancy.. Eur JPediatr.

[17] von Kries R, Toschke AM, Koletzko B, Slikker W (2002). Maternal smoking during pregnancy and childhood obesity.. Am J Epidemiol.

[18] Montgomery SM, Ekbom A (2002). Smoking during pregnancy and diabetes mellitus in a British longitudinal birth cohort.. BMJ.

[19] Power C, Jefferis B (2002). Fetal environment and subsequent obesity: a study of maternal smoking.. Int J Epidemiol.

[20] Grassi G, Arenare F, Quarti-Trevano F, Seravalle G, Mancia G (2009). Heart rate, sympathetic cardiovascular influences, and the metabolic syndrome.. Prog Cardiovasc Dis.

[21] Al Mamun A, Lawlor DA, Alati R, O'Callaghan MJ, Williams GM (2006). Does maternal smoking during pregnancy have a direct effect on future offspring obesity? Evidence from a prospective birth cohort study.. Am J Epidemiol.

[22] Najman JM, Bor W, O'Callaghan M, Williams GM, Aird R (2005). Cohort Profile: The Mater-University of Queensland Study of Pregnancy (MUSP).. Int J Epidemiol.

[23] Keeping JD, Najman JM, Morrison J, Western JS, Andersen MJ (1989). A prospective longitudinal study of social, psychological and obstetric factors in pregnancy: response rates and demographic characteristics of the 8556 respondents.. Br J Obstet Gynaecol.

[24] World Health Organization (1997). Obesity. Preventing and Managing the Global Epidemic. Report of a WHO Consultation on Obesity, 3–5 June 1997. Geneva, Switzerland.. : World health Organization, 1998.

[25] World Health Organization (2000). Obesity: preventing and managing the global epidemic. Report of a WHO consultation.. World Health Organ Tech Rep Ser;894:i–xii, 1–253.

[26] Mackinnon DP, Dwyer JH (1993). Estimating mediated effects in prevention studies.. Evaluation Rev.

[27] Swauger JE, Steichen TJ, Murphy PA, Kinsler S (2002). An analysis of the mainstream smoke chemistry of samples of the U.S. cigarette market acquired between 1995 and 2000.. Regul Toxicol Pharmacol.

[28] Rustemeier K, Stabbert R, Haussmann HJ, Roemer E, Carmines EL (2002). Evaluation of the potential effects of ingredients added to cigarettes. Part 2: chemical composition of mainstream smoke.. Food Chem Toxicol.

[29] Gao YJ, Holloway AC, Zeng ZH, Lim GE, Petrik JJ (2005). Prenatal exposure to nicotine causes postnatal obesity and altered perivascular adipose tissue function.. Obes Res.

[30] Kane JK, Parker SL, Matta SG, Fu Y, Sharp BM (2000). Nicotine up-regulates expression of orexin and its receptors in rat brain.. Endocrinology.

[31] Slotkin TA (1998). Fetal nicotine or cocaine exposure: which one is worse?. J Pharmacol Exp Ther.

